# A Structural Perspective of the Role of IP6 in Immature and Mature Retroviral Assembly

**DOI:** 10.3390/v13091853

**Published:** 2021-09-17

**Authors:** Martin Obr, Florian K. M. Schur, Robert A. Dick

**Affiliations:** 1 Institute of Science and Technology (IST) Austria, 3400 Klosterneuburg, Austria; martin.obr@ist.ac.at; 2 Department of Molecular Biology and Genetics, Cornell University, Ithaca, NY 14853, USA

**Keywords:** HIV, inositol hexakisphosphate, orthoretrovirus, Gag

## Abstract

The small cellular molecule inositol hexakisphosphate (IP6) has been known for ~20 years to promote the in vitro assembly of HIV-1 into immature virus-like particles. However, the molecular details underlying this effect have been determined only recently, with the identification of the IP6 binding site in the immature Gag lattice. IP6 also promotes formation of the mature capsid protein (CA) lattice via a second IP6 binding site, and enhances core stability, creating a favorable environment for reverse transcription. IP6 also enhances assembly of other retroviruses, from both the Lentivirus and the Alpharetrovirus genera. These findings suggest that IP6 may have a conserved function throughout the family Retroviridae. Here, we discuss the different steps in the viral life cycle that are influenced by IP6, and describe in detail how IP6 interacts with the immature and mature lattices of different retroviruses.

## 1. Introduction

**Figure 1:**
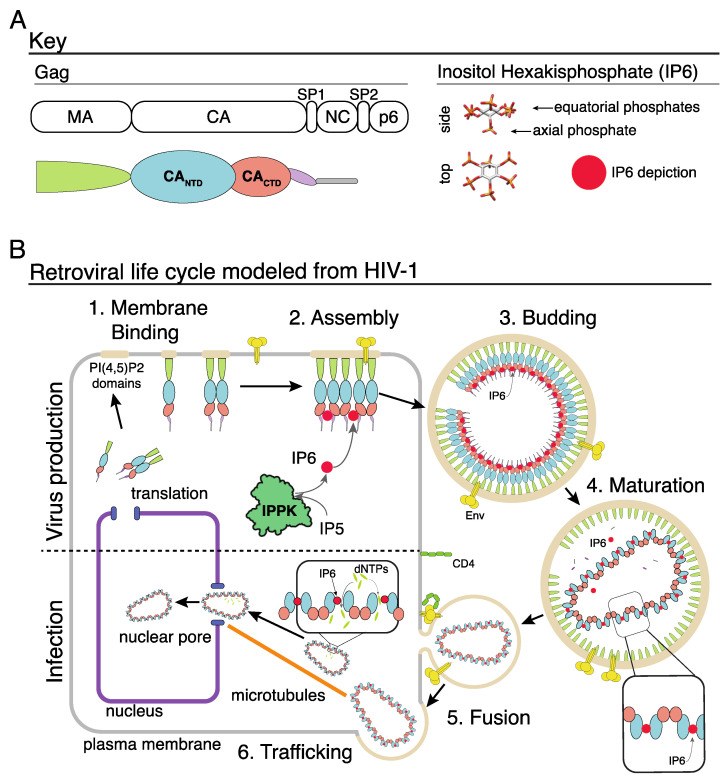
IP6 and the retroviral life cycle. (**A**) Key of the HIV-1 Gag domains on left. Right shows the d-myo-IP6 molecule. (**B**) 1: Gag protein interacts with the inner leaflet of the cellular plasma membrane. 2: Interactions between Gag, the plasma membrane, nucleic acid, and IP6 result in the assembly of the immature Gag lattice. 3: Assembled virion buds from cell with IP6 bound to two rings of six lysine residues at the Gag hexamer interface. 4: Retroviral protease cleaves the Gag protein, resulting in the liberation of the CA domain. CA interacts with IP6 via a ring of six arginine residues in the CA_NTD_ hexamer interface, which leads to the formation of the mature core. 5: Interaction between the viral Env protein and the cell receptor (CD4 in this example) results in fusion, and release of the viral core into the cell cytoplasm. 6: Trafficking of the viral core along microtubules to the nuclear pore. Once in the cytoplasm, dNTPs enter the capsid core where they “feed” reverse transcription of the viral RNA genome into double-stranded DNA. Once at the nuclear pore, or inside the nucleus, the capsid core breaks open, releasing the integration complex.

All retroviruses code for a multidomain structural protein Gag (Figure 1A). Late in the viral life cycle, Gag traffics to the inner leaflet of the plasma membrane where the N-terminal Gag matrix (MA) domain interacts with the local lipid environment (Figure 1B, step 1 and 2). Specific binding of the C-terminal Gag nucleocapsid (NC) domain to the viral genomic RNA stimulates assembly and ultimately leads to incorporation of the genome into the budding virus. Protein–protein interactions between the bipartite capsid domains (consisting of CA_NTD_ and CA_CTD_) in Gag result in formation of the immature hexameric Gag lattice. Following release of the immature virus particle from the infected cell (Figure 1B, step 3), the viral protease (PR) cleaves Gag, liberating CA and other Gag domains (Figure 1B, step 4). In this process, termed maturation, all immature CA–CA interactions are broken and an entirely new set of interactions between CA proteins form the mature lattice, consisting of CA hexamers and pentamers, resulting in the mature core (also referred to as the capsid). Retrovirus assembly and maturation are tightly regulated processes, in which ordered assembly and complete proteolytic cleavage of Gag in the correct temporal sequence are required for the virus to gain infectivity [1,2]. For more details on retrovirus assembly and maturation, we refer the reader to other excellent reviews on this topic [3].

In the case of HIV-1, after the infecting virus fuses with the cell membrane, the released mature core is trafficked along microtubules to nuclear pores (Figure 1B, steps 5 and 6) [4]. Reverse transcription of the viral RNA occurs within the core, which provides a protected environment for the viral genome, while at the same time sets preconditions for the required import of nucleotides into the core interior [5]. Recent literature has revealed the involvement of the small negatively charged molecule inositol hexakisphosphate (IP6) in all of these steps of the HIV-1 life cycle, supporting its key structural and functional role in regulating HIV-1 assembly, maturation, and reverse transcription. Specifically, in HIV-1, IP6 is essential for the formation of the immature lattice, where it is coordinated in the center of the CA_CTD_ hexamer by two rings of six lysine residues. In the mature CA lattice, IP6 acts at the intrahexamer interface of six arginines residues near the CA N-terminus.

In this review, we summarize the current knowledge of the essential functions of IP6 in the early and late phases of the viral life cycle, specifically focusing on the work that elucidated the structural aspects of IP6 binding and stabilization of both the immature Gag lattice and subsequently the mature CA lattice. We also discuss the importance of IP6 for other retrovirus genera, indicating an evolutionarily conserved structural role of this molecule.

## 2. Immature Lentivirus Gag Assemblies Uniquely Coordinate IP6

IP6 has been the subject of numerous studies analyzing its interaction with Gag. Twenty years ago, inositol phosphates were reported to stimulate assembly of structurally authentic immature virus-like particles (VLPs) from full-length Gag molecules. Initially, it was suggested that the binding of IP6 occurs in the domains with high concentration of positively charged residues—MA and NC [6]. These conclusions were supported by observations showing that Gag proteins with deletions of residues 16–99 in the MA domain were not stimulated by IP6 under conditions where full-length Gag was [6]. Indeed, this was later confirmed as IPs were found to bind to Gag at both the N-terminal MA and C-terminal NC domains [7,8]. A more recent study showed incubation of HIV MA with IP6 increased MA trimerization and MA interaction with the Env glycoprotein cytoplasmic tail [9]. However, the precise interaction between IP6 and MA and NC in the context of the Gag lattice remained enigmatic.

In recent years, significant progress has been made in our understanding of retrovirus assembly by studying either immature and mature virus particles or VLPs assembled from purified proteins in vitro, via cryo-electron tomography (cryo-ET) and subtomogram averaging (for more details on these methods, we refer the readers to exhaustive reviews on these topics [10,11,12]. A variety of methodological advancements in microscope instrumentation, data acquisition, and image processing software (for examples, see [13,14,15,16]) resulted in high resolution of such complex and pleomorphic virus assemblies, allowing unambiguous interpretations of the CA–CA interactions forming the immature Gag lattice, which in case of lentiviruses, remains incomplete at the site of scission during budding [17]. For HIV-1, these studies revealed important inter- and intrahexamer CA interaction interfaces formed by both the CA_NTD_ and CA_CTD_ domains [18], and also revealed the role of the six-helix bundle formed by the CA_CTD_–SP1 helix in stabilizing the immature hexamer and regulating proteolytic maturation [19]. In particular, the resolution obtained for the immature HIV-1 Gag assemblies was sufficient to model the CASP1 backbone and large side chains. Specifically, 12 lysine residues (K290 in the MHR loop upstream of helix 8 in CA_CTD_ and K359 in the CA_CTD_–SP1 helix) coordinate a prominent density in the center of the immature hexamer. The identity of this density remained unassigned in this first study but was suggested to be a negatively charged ion cluster [19].

**Figure 2:**
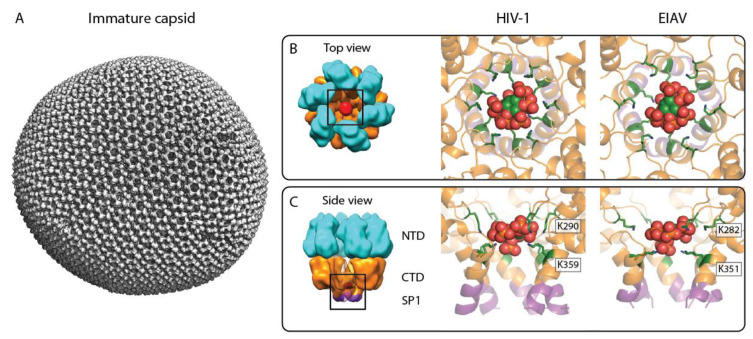
IP6 binding site in the immature retroviral CA lattice. (**A**) Composite isosurface representation of an immature retroviral CA lattice derived from an EIAV VLP, representative of the immature CA arrangement in the lentivirus genus. Please note that in authentic immature lentivirus particles, the Gag lattice is incomplete. (**B**,**C**) Top and side view of the IP6 binding site in the immature CA hexamer, respectively. Left: cartoon model of an immature CA hexamer; cyan: CA_NTD_, orange: CA_CTD_, purple: SP1, and red: IP6. Middle: X-ray crystallographic model of IP6 binding to the immature HIV-1 CA_CTD_–SP1 hexamer (pdb: 6BHR). Right: model of IP6 binding to the immature EIAV CANC hexamer, based on a structure solved by cryo-ET and subtomogram averaging (pdb: 6T64). The color coding for CA_CTD_–SP1 is as in the left panel. The IP6 molecule is shown as sphere representation in green, orange, and red. The lysines coordinating the IP6 molecule are shown in a green stick representation and are annotated.

Structural, biochemical, and cellular experiments with HIV-1 Gag proteins then unambiguously demonstrated that the density coordinated by residues K290 and K359 in native virus is IP6 [20]. A structure of HIV-1 CA_CTD_–SP1 crystal grown in the presence of IP6 clearly showed that the molecule on the rotation axis of the CA hexamer is coordinated by the two rings with a total of 12 lysines [20] (Figure 2B,C). The IP6 density in the crystal structure is compatible with its most stable conformation—myo-IP6—with the one phosphate in the axial position, coordinated by the K359 ring, and the other five in the equatorial positions, coordinated by the K290 ring.

Mutational analyses have shed further light on the functions of IP6 in the immature Gag lattice. Replication of HIV-1 carrying either a Gag K359 mutation or a K290 mutation is severely decreased [20,21]. By selection for recovery of infectivity, it was found that Gag with mutation T371I (residue 8 in the SP domain) suppresses the K359 defect [21]. This mutation was described previously as stabilizing the six-helix bundle [22]. However, the double mutant HIV-1 (Gag K359A, T371I) still packages IP6 [21]. One possible explanation is that the K359 interaction with the axial phosphate of IP6 is required for six-helix bundle stabilization. In the absence of this interaction (i.e., K359A), the stabilizing mutation in SP is required. However, these changes do not alter the MHR K290 interaction with the five equatorial phosphates.

IP6, and to a lesser extent inositol phosphates with fewer phosphate groups, is more favorable to immature lattice formation, acting like a switch in HIV-1 CASPNC in vitro assembly [23], from a mature to an immature lattice. Replacing residues K290 and K359 with alanines abrogates inositol phosphate responsiveness. Since the CASPNC protein employed in these experiments lacks the MA domain, an assembly effect of IP6 via the N-terminal end of Gag can be ruled out. Remarkably, the minimal CASP1 protein in presence of IP6 also assembles into VLPs with a lattice consistent with an immature arrangement of CA [20]. Assembly of CASP1 under these conditions represents one of just a few examples of immature assembly in the absence of NC or nucleic acid [24,25].

As mentioned above, the position of the IP6 molecule in the CA_CTD_–SP1 crystal coincides with the density observed in cryo-ET structures obtained from purified immature HIV-1 [18,19]. A similar density also was found in a sub-4 Angstrom structure of the immature CASP1 lattice obtained from an in vitro assembled, *E. coli*-expressed truncated Gag protein missing most of the MA domain (amino acids 16–99) [19]. Since *E. coli* does not contain IP6, and no additional IP6 was added to the assembly reaction, other molecules presumably also can be coordinated by the 12 lysines [26]. In any case, in the absence of a negatively charged molecule that neutralizes the two rings of twelve lysines, immature assembly appears not to occur. This conclusion is in accordance with the interpretation that the main effect of IP6 binding is charge compensation, a role that could in principle be taken by other negatively charged molecules. Indeed, the structurally similar, but not biologically relevant, molecule hexacarboxybenzene (mellitic acid) promotes limited immature virus-like particle assembly of HIV-1 CANC protein [20]. In contrast, dNTPs do not influence immature assembly [20], suggesting that while charge compensation is important, the positioning of charges within the molecule is also relevant.

Originally, CACTDSP1 protein was found to be refractory to crystallization in an immature-like form. However, a seminal study by Wagner and colleagues [27] was successful in identifying appropriate crystallization conditions. In this study, the authors were able to obtain a high-resolution structure of parts of the immature lattice, at the same time as identical results were obtained by cryo-ET and subtomogram averaging in native virus particles [19]. The compound that was required for crystallization was bis-tris propane, a 2-fold symmetric molecule with negative hydroxyl groups at either end. The dimension of this compound along its long axis is similar to that of IP6. The dynamic behavior of the CA_CTD_–SP1 hexamer and the importance of IP6 in stabilizing it were further exemplified by molecular dynamics (MD) simulations [20]. Again, in line with the shown potential of other inositol derivatives or mellitic acid to promote immature HIV-1 CANC assembly, these molecules led to a clear stabilization of the CASP1 helix and hence the immature CASP1 hexamer.

A role of IP6 in lentiviruses other than HIV-1 was then shown in a study focusing on equine infectious anemia virus (EIAV), which is distantly related to HIV, separated by ~100 million years [28]. While it was previously observed that the quaternary CA assembly between different retrovirus genera can substantially differ [1,18,29,30,31], the comparison of EIAV and HIV immature Gag assemblies demonstrated that within the Lentivirus genus, the immature CA assembly is largely conserved [32]. The sub-4 Å structures of EIAV CANC VLPs obtained by cryo-ET and subtomogram averaging revealed similar inter and intrahexameric CA interactions in EIAV and HIV-1, involving trimeric CA_NTD_ contacts as well CA_CTD_ interactions around the hexameric ring, respectively. Notably, EIAV also forms a 6HB consisting of the last residues of CA and the first residues of SP, which is slightly shorter than its HIV-1 counterpart.

The EIAV CASP model also showed IP6 binding identical to that in HIV-1, with two lysine rings (K282 and K351 in EIAV Gag, see Figure 2) being stacked on top of each other and coordinating an IP6 molecule in its myo-conformation in the center. Biochemistry and mutagenesis experiments, where the lysines were mutated to alanines, underscored the conserved role of IPs in the immature assembly within the Lentivirus genus. However, in contrast to HIV-1, molecular dynamic simulation studies on the EIAV CASP hexamer showed that IP6 is not required to stabilize the six-helix bundle [32]. While the binding of IP6 to the lysine-coordinated position was stable, the stability of the 6HB in the absence of IP6 was conferred by the stronger hydrophobic side chain interactions between the 6HB helices.

**Figure 3:**
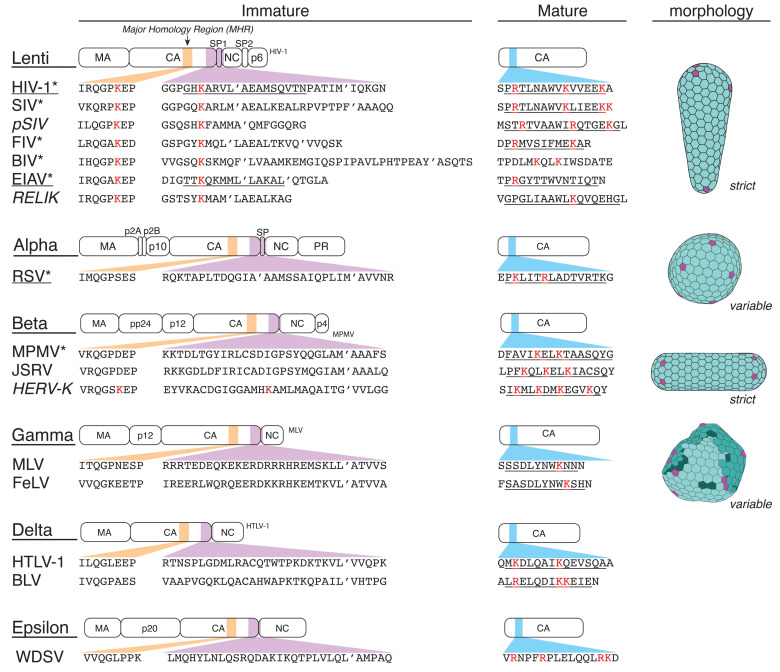
Retrovirus sequence comparison. The immature IP6 binding site in Lentiviruses in the MHR and CA_CTD_SPNC region (immature) and CA helix 1 (mature). Lys (K) and Arg (R) residues that are at or near known IP6 binding sites are in red. Simplified morphologies shown are on the right; conical, spherical, cylindrical, nested cores (layering). A minimum of one sequence is displayed for each retrovirus. * = retroviruses with reported IP6-related phenotypes. Italics = endogenous retroviruses. Underlined = retroviruses with solved structures with an IP6 interaction. Underlined amino acid sequences correspond to known alpha-helical structures.

Beyond HIV-1 and EIAV, no other immature lentivirus structures from Gag assemblies are available. However, it is reasonable to assume that the striking structural feature of 12 lysines coordinating a negatively charged molecule is widely conserved within lentiviruses, as conserved lysine residues can be observed at identical positions in Gag of other members of this genus, including HIV-2, SIV, FIV, and BIV (Figure 3). Experimental support for this hypothesis was provided by in vitro assembly experiments using Gag-derived truncation variants of these viruses, where IP6 had a significant stimulatory effect [32]. IP6 has been speculated to have an influence on the immature assembly of Rous Sarcoma virus (RSV), based on assembly kinetics in vitro [33]. However, this effect has so far not been explained in structural terms.

At the moment, no data indicate that the immature assembly of retrovirus species from genera other than Lentivirus and Alpharetrovirus is IP6-dependent, as they do not contain positively charged residues in the same positions within Gag (Figure 3). Moreover, the available structures of immature Gag assemblies of the Alpharetrovirus RSV [29], the Betaretrovirus M-PMV [18,30], or the Gammaretrovirus MLV [31] do not show a similar structural site that would coordinate an IP6-like density. This could mean either that there is an alternative binding site in other Gag domains, or that IP6 is dispensable for immature assembly of these viruses.

## 3. IP6 Enhances Key CA Properties Required for Infectivity

In immature Gag assembly, the quaternary Gag protein arrangement can vary because of different CA_NTD_ interactions, while in mature CA assembly the CA–CA interactions appear well conserved in different retrovirus genera [1,31,34,35,36,37,38]. However, mature CA cores display a remarkable polymorphism, where the architectures from different virus species (and even within one species) can vary significantly (Figure 3, morphology panel). Thus, different retroviruses can display conical CA cores (e.g., HIV-1), irregular multilayered cores (MLV), polyhedral cores (RSV), tubular cores, and closed cylindrical cores (M-PMV, RSV). As shown for HIV-1, the stability of the mature core is a very tightly balanced parameter that is critical for infectivity [39,40,41]. On the one hand, the core of HIV-1 must be stable enough for trafficking through the cytoplasm to the nucleus, to protect the viral genome during reverse transcription and to allow for dNTP import as a substrate for reverse transcription. On the other hand, it must be able to disassemble to allow integration of the proviral DNA into a chromosome.

As discussed, upon maturation and proteolytic cleavage, CA–CA interactions within the immature Gag lattice are broken or rearranged and new interactions are formed to build the mature CA core [1]. This structural maturation also involves release of IP6 from the immature CASP coordination site. IP6 is then available to act to promote assembly of and stabilize the mature CA core.

Similar to the immature HIV-1 CASP1 assembly, the mature HIV-1 CA hexamer contains a ring of positively charged residues (R150 in Gag, R18 in CA—here referred to as R18) that coordinate IP6 in the hexamer center. Moreover, the center of the CA hexamer was designated to be a pore important for dNTP import and reverse transcription [42]. An earlier study had previously proposed a role of arginine clusters in protein function and assembly [43], by suggesting that clustered arginines could be involved in charge compensation of negatively charged counter ions.

In 2018, the same study that first reported the structural role of IP6 in the immature assembly also reported how IP6 regulates mature HIV-1 capsid assembly and stability [20]. Specifically, IP6 stimulates high efficiency in vitro *CA* assembly, which is lost upon mutation of R18 to alanine. Infectivity data also revealed that the R18A mutation leads to reduced infectivity; however, this effect likely is due to a reduced virus production [44]. In line with these results, it was also shown that IP6 can stabilize capsid cores isolated from cells [39,40,45,46].

**Figure 4:**
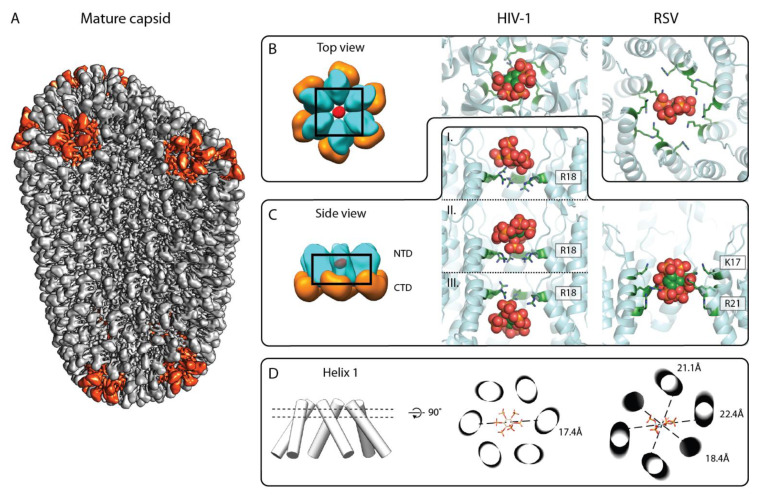
IP6 binding site in the mature retroviral CA lattice. (**A**) Composite isosurface representation of the mature CA lattice forming a conical core. Adapted from [37]. Grey: CA hexamers, red: CA pentamers. (**B**,**C**) Top and side view of the IP6 binding site in the mature CA hexamer, respectively. Left: schematic model of mature HIV-1 CA hexamer; cyan: CA_NTD_, orange: CA_CTD_, and red: IP6. Middle: X-ray crystallographic model of IP6 binding to mature HIV-1 CA hexamer (6BHS, top view). IP6 has been reported to bind at different heights of the HIV-1 CA_NTD_ pore, based on different crystal structures, shown in (**C**) (pdb: 6BHT (top and bottom), 6BHS (middle)). Right: model of IP6 binding in the RSV CANC hexamer, derived from a structure solved by cryo-ET and subtomogram averaging (pdb: 7NO2). Note the orientation of the IP6 molecule with four axial phosphates pointing up or down, and one axial and the equatorial phosphate pointing horizontally. The color coding for CA_NTD_ is as in the left panel. The IP6 molecule is shown in sphere representation in green, orange, and red. The arginines and lysines coordinating the IP6 molecule are shown in a green stick representation and are annotated. (**D**) Helix 1 orientation with respect to IP6 binding. Left: cylinder model of the central pore formed by helix 1. Middle and right: cross-section through helix 1 cylinders at the height of IP6 binding plane for the C6 symmetric HIV-1 CA hexamer (as seen in the middle panel of B) and the C2 symmetric RSV CA hexamer (as seen in the right panel of B), respectively. IP6 is shown as a stick model. Dashed lines show distances between the opposite Cα atoms of R18 of HIV-1 CA in the middle image and K17 of RSV CA in the right image, and emphasize the deviation of the RSV hexamer from sixfold symmetry.

A crystal structure confirmed the expected IP6 binding site in the mature HIV-1 CA hexamer, where IP6 was found in the central pore coordinated by the six R18 sidechains [20]. However, in one of two solved crystals, not one but two densities for IP6 were observed, indicating that IP6 can be situated above or below this ring of basic residues formed by R18 (Figure 4B,C). These structural observations of IP6 binding in the mature HIV-1 CA hexamer were independently confirmed by others [40,44,47] (Figure 4C).

Based on structural data, IP6 appears to have the greatest number of interactions when bound above the R18 ring. A density in the same position had been observed earlier in authentic mature HIV-1 particles [48]. However, the resolution of the structure from authentic HIV-1 virions was too low to unambiguously identify the molecule, and hence the density in the mature CA pore could have been due to other charge-compensating molecules. Specifically, the pore formed by helix 1 has been proposed to mediate transport of negatively charged molecules into the mature core [42], and different polyanions have been captured by X-ray crystallography in the center of CA hexamer, e.g., mellitic acid, dNTPs, or a reverse transcriptase inhibitor [40,42].

Below R18, a second ring of basic residues is formed by lysines (K25 in HIV-1 CA). K25 mutations result in reduced infectivity [49], reduced reverse transcriptase activity, and maturation defects [47]. All-atom MD simulations (MD) for HIV-1 pentamers and hexamers [50] revealed how the presence of these two rings of basic residues can coordinate IP6 in different binding modes. While hexamers could stably bind IP6 molecules at two positions, either above or below the R18 ring, pentamers could coordinate only one IP6 molecule above the basic residues. These simulations, which characterized the binding of IP6 to preformed mature CA hexamers and pentamers, and the co-assembly of CA and IP6, also suggested that IP6 stabilizes pentamers more strongly than it does hexamers, and therefore could shift assembly conditions towards pentamers. This prediction is consistent with the early observation that low concentrations of IP6 stimulate CA assembly into tubes (which are made primarily of hexamers), while high concentrations of IP6 stimulate assembly of polyhedrons (which must contain pentamers) [20]. The role of K25 in dNTP import was also analyzed in more detail by all-atom MD simulations [46] and later by encapsidated reverse transcription experiments, in which the RT activity of purified capsid cores from pseudotyped virus is assayed as a measure of dNTP import and core stability [47].

In immature retrovirus Gag assemblies, the positively charged residues required to coordinate IP6 in the hexamer center seem to be predominantly found in the Lentivirus genus. In contrast, a putative IP6 binding site in mature CA cores appears to be conserved among different retroviral genera. Specifically, positively charged residues (either lysines or arginines, or both) are present within helix 1 (or adjacent regions) of almost all orthoretroviruses (Figure 3). Proof for such a conserved role of IP6 outside the lentiviruses genus was recently provided by a study on the mature alpharetrovirus RSV. A structure of the mature RSV CA hexamer solved from CANC tubes, which were assembled in vitro in the presence of IP6, showed a density in the center of the hexamer pore [37], where it is coordinated by a ring of six lysines (K17 in RSV CA) and six arginines (R21 in RSV CA) (Figure 4B–D right panel). The density suggested that the IP6 molecule is oriented “on edge” with the equatorial phosphates pointing up and down in the pore, which contrasts with the “flat” orientation of IP6 in HIV-1 CA.

One explanation for the different orientation of IP6 in mature RSV could be that the RSV CA hexamer is more flexible than the HIV CA hexamer, and the central pore is wider than in HIV-1 (Figure 4D). The implementation of classification and alignment workflows to address the increased structural pleomorphism of RSV cores [37], which can form polyhedrons, cones, or tubes [51], showed that the RSV CA hexamer substantially deviates from C6 symmetry. This results in a deformation of the central pore, manifested as increased and uneven separation of helices 1 from different CA molecules (Figure 4D). This deformation allows the small IP6 molecule to be captured inside the lysine ring in an upright position, unlike the binding observed in HIV-1 above and below the R18 pore. Experiments in RSV also showed that phosphate promotes pentamer formation, while inositol phosphates promote hexamer formation. Such differential mechanisms of charge compensation in RSV could be related to the varying flexibility of hexamers over pentamers, where the latter have been found to be structurally rigid organization centers within the mature CA lattice [37].

As we and others have hypothesized [5], HIV selectively recruits the cytosolic molecule IP6 into virions via its immature Gag IP6-binding site, thus increasing the local concentration of IP6 within virions and promoting formation of a mature lattice following Gag cleavage. Consistent with the absence of basic residues near or at the MHR and the putative 6HB of RSV, IP6 did not enhance immature RSV assembly [37]. This observation suggests that IP6 is not selectively recruited into immature RSV particles, and also agrees with the finding that RSV requires significantly lower IP6 concentrations than does HIV-1 to promote mature assembly [37]. Thus, one may speculate that other retroviruses lacking basic residues for an immature IP6 binding site, but having conserved basic residues at the mature CA_NTD_ position, could also use IP6 to regulate mature core assembly and maturation. Whether nonspecific incorporation of IP6 into the assembling virus is sufficient to promote mature assembly needs to be addressed in future studies.

## 4. IP6 in Infected Cells Is Essential for HIV-1 and RSV Replication

How do these structural effects of IP6 binding to and stabilizing the immature and the mature viral lattice relate to IP6 in cells? As already outlined in the above sections, mutations of residues involved in IP6 coordination have a significant impact on viral replication and spread. An alternative approach taken by multiple research groups has been to modify IP6 (and IP5) levels in cells by knocking out the enzymes that convert IPs to IP6, or to transiently express enzymes that ablate IP6 [32,37,44,52,53], or to carry out recovery assays in which kinases required for IP6 synthesis are added back to KO cell lines [52].

In mammalian cells, IP6 concentrations are reported to range from 10 to 100 µM [54]. The last step in the biosynthetic pathway leading to IP6 is the phosphorylation of IP5 by the enzyme IPPK [55,56,57,58] (Figure 1). Ablation of the IPPK gene by CRISPR-Cas9 knockout reduces IP6 to nondetectable levels. When infected with HIV-1, these IPPK-KO cells fail to release virus particles, demonstrating that IP6 is required for immature virus particle formation [20,21,32,37,52,53]. The effect of IP6 removal is not cell type-dependent, since HIV-1 production was found to be highly diminished both in MT4 and in HEK 293T cells when the IPPK gene was ablated [53]. Consistent with these results, a pulse-chase experiment in which virus was collected from ^3^H-inositol-labeled cells, contained radiolabeled IP6 calculated to be at levels of 309 (plus or minus 41) molecules per virion, corresponding to approximately one IP6 molecule per Gag hexamer [40]. Other lentiviruses are similarly dependent on IP6. Simian immunodeficiency virus (SIV) and feline immunodeficiency virus (FIV), and EIAV all demonstrate a loss in infectious virus particle release when IP6 synthesis is blocked [44,52]. Beyond the Lentivirus genus, RSV also requires IP6 for efficient infectious virus particle release from cells [37].

Beyond its critical structural role in stabilizing the mature CA lattice, IP6 may also be critical in the early phases of the virus replication cycle. A long-held model posits that the HIV capsid cracks open in the cytoplasm, and dNTPs enter the core through these cracks. However, recent evidence clearly shows that the intact capsid core enters the nucleus through nuclear pores [4], where the integration complex (viral DNA and integrase) is released from the core, and integration of the viral genome into the host genome occurs. This would require that the capsid core remains intact during reverse transcription. Recent studies suggest that during trafficking, dNTPs from the cytoplasm enter the capsid core where they feed reverse transcription of the viral RNA into first single-stranded and then double-stranded DNA [39,40,46]. All-atom MD simulations demonstrate that dNTPs can enter the capsid core via the hexamer pore formed by the ring of six R18 residues, and that the presence of IP6 facilitates dNTP import [46]. This model is supported by in vitro experiments demonstrating that capsid core stability and reverse transcription are enhanced by the presence of IP6 [39,40,46]. Taken together, these observations provide an updated picture of both early and late effects on HIV-1 replication.

## 5. Conclusions and Future Directions

Since the elucidation of the structural aspects of IP6 binding to immature Gag and mature CA lattices, numerous follow-up studies have further characterized how this small molecule regulates the retroviral life cycle at numerous steps. While initially observed for HIV-1, increasing evidence shows that IP6 plays an evolutionarily conserved role in the Lentivirus genus and, at least to some extent, also in other orthoretroviruses.

Exciting directions remain to be explored: For example, why did HIV-1 and other retroviruses develop and maintain such a peculiar dependence on a charge-compensatory molecule like IP6? Future work should also examine if IP6 plays a role in even more distantly related exogenous and endogenous retroviruses, and in DNA and RNA viruses of other taxa.
